# Modeling the Spatiotemporal Dynamics of Oncolytic Viruses and Radiotherapy as a Treatment for Cancer

**DOI:** 10.1155/2020/3642654

**Published:** 2020-04-12

**Authors:** Eman Simbawa, Najwa Al-Johani, Salma Al-Tuwairqi

**Affiliations:** Department of Mathematics, Faculty of Science, King Abdulaziz University, Jeddah 21589, Saudi Arabia

## Abstract

Virotherapy is a novel treatment for cancer, which may be delivered as a single agent or in combination with other therapies. Research studies indicated that the combination of viral therapy and radiation therapy has synergistic antitumor effects in *in vitro* and *in vivo*. In this paper, we proposed two models in the form of partial differential equations to investigate the spatiotemporal dynamics of tumor cells under virotherapy and radiovirotherapy. We first presented a virotherapy model and solved it numerically for different values of the parameters related to the oncolytic virus, which is administered continuously. The results showed that virotherapy alone cannot eradicate cancer, and thus, we extended the model to include the effect of radiotherapy in combination with virotherapy. Numerical investigations were carried out for three modes of radiation delivery which are constant, decaying, and periodic. The numerical results showed that radiovirotherapy leads to complete eradication of the tumor provided that the delivery of radiation is constant. Moreover, there is an optimal timing for administering radiation, as well as an ideal dose that improves the results of the treatment. The virotherapy in our model is given continuously over a certain period of time, and bolus treatment (where virotherapy is given in cycles) could be considered and compared with our results.

## 1. Introduction

Cancer is a complex disease, and its complexity causes it to resist conventional therapies [1]. Accordingly, the combination of different cancer therapies emerged as one of the novel strategies that are aimed at improving the outcome (killing cancer). The principle of combination therapies consists of the use of various attack mechanisms that prevent cancer from resisting treatment [2]. Recently, oncolytic viruses have been vigorously investigated as an anticancer treatment. Virotherapy is used either as a single agent or in combination with different conventional cancer therapies such as chemotherapy [3] and radiation therapy [4].

Virotherapy destroys tumor cells through a mechanism different from radiation therapy. Although the mechanisms of oncolytic viruses are not entirely obvious, these viruses have the ability to selectively target, replicate in, and destroy cancer cells [5]. In contrast, the radiation therapy destroys the cancer cells by directly damaging the DNA or indirectly, by forming oxygen radicals which disturb cellular pathways [2]. Furthermore, the combination of these two therapies leads to synergistic relationships, as radiation may enhance viral uptake, viral gene expression, and viral replication [4].

Numerous research studies have demonstrated that the combination of oncolytic viruses and radiotherapy leads to promising therapeutic results that are not attainable by monotherapy [6–9]. For example, the study by Bieler et al. [7] determined that utilizing the oncolytic adenovirus dl520 in conjunction with radiation therapy resulted in an increase in viral replication. They concluded that the combination of radiation and dl520 achieved the inhibition of tumor growth by 89% after 32 days from the beginning of treatment. This is significantly more effective than giving each treatment separately, where the tumor growth was inhibited by dl520 and radiation by 45% and 52%, respectively. Another experimental study carried out by Dilley et al. [8] indicated that the oncolytic adenovirus CG7870 and radiotherapy, when combined, lead to increased antitumor efficacy with lower doses. Furthermore, their results showed that this combination resulted in a significant mean tumor volume that is 34% of the baseline, 39 days after treatment. However, when considering CG7870 and radiation as separate treatments, the mean tumor volume reached 121% and 126% of the baseline, respectively.

Establishing mathematical models is an effective tool to gain more insights concerning combined treatments. Dingli et al. [10] developed a mathematical model in the form of ordinary differential equations (ODEs), for cancer radiovirotherapy treatment. This model has equilibrium points showing complete and partial eradication of cancer in addition to therapy failure. In addition, Tao and Guo [11] generalized the model in [10] by considering the spatiotemporal distribution of tumor cells. By numerical simulations, they concluded that radiovirotherapy is more effective than virotherapy alone. They also indicated that there is an optimal timing of radioiodide administration and an optimal dose of the radioactive iodide that could achieve favorable results with this treatment.

Jenner et al. [12] introduced a mathematical model consisting of three nonlinear ODEs describing the interaction between tumor cells and oncolytic virus therapy. In this paper, we will develop these ODEs by considering the spatial variation and diffusion of cancer cells. Thus, we include a diffusion term for the tumor cell density as well as virus density (we assume that viruses diffuse into cancer). In addition, we will add a term describing the elimination of free viruses due to the infection of tumor cells. This is based on the assumption that when a virus infects a cancer cell, it becomes inactive and cannot infect other cancer cells [13]. Thus, it cannot be considered as a part of the free virus population. In addition to this model, we will introduce another model where we include the effect of radiotherapy in combination with virotherapy. The aim of this paper is to predict the outcome of two phases of treatments where virotherapy is given alone for a certain period of time (Model 1, Phase I); then immediately afterwards, radiotherapy is introduced in conjunction with virotherapy (Model 2, Phase II). Thus, the two models will be solved numerically with different values for the parameters along with various doses of radiation (Model 2) to determine the optimal strategy that can produce the ideal results for the treatment. This paper is organized as follows: In Section 2, we present a model for virotherapy and solve it numerically with different values for the parameters, where the virus is delivered to the tumor continuously. In Section 3, we extend the virotherapy model by including the effect of radiotherapy. Afterwards, we numerically study it with three modes of the radiation delivery which are constant, decaying, and periodic (similar to the model in [14]).  Section 4 will conclude with a discussion and suggestions for further research.

## 2. Phase I: Virotherapy Treatment

### 2.1. Model Description

To further understand the effects of viral therapy on tumor growth, we present a model describing the dynamic interactions between tumor cells and viral therapy. Our model is formulated in terms of partial differential equations (PDEs) that take into consideration the spatiotemporal variation of tumor cells. This model represents Phase I of the treatment for cancer.

We assume that tumor cells depend on the closest blood vessel. For simplicity, we assume that the region is cylindrically symmetric, and thus, the variables depend on time *t* and radial distance *r*. Moreover, we estimate the radius of this region supported by the blood vessel to be $r_{b}/\sqrt{\text{BVF}}$, where BVF is the blood volume fraction [15, 16] and *r*_*b*_ is the radius of the blood vessel. Regarding the viruses, they reach the tumor via the blood vessel and diffuse into it with no flux at the boundary. Furthermore, viruses are given continuously with a constant concentration *v*_0_ for a certain time interval (for example by using nanotechnology [17]). Also, we consider no flux for the uninfected and infected tumor cells at the blood vessel wall and at the boundary of the cylinder.

Suppose that when oncolytic viruses reach the tumor through the blood vessel, they diffuse into it and infect some of the cells. Therefore, the tumor cells can be divided into uninfected and infected cells. We assume that the tumor is aggressive which means that it replicates proportional to its size (uninfected cancer cells grow exponentially). To model the movement of uninfected and infected tumor cells, we include a diffusion term in their equations. In addition to this, viruses replicate inside the infected tumor cells, which causes lysis to them. This leads to the release of new virus particles, which infect other tumor cells. As a result of this, we assume that viruses are removed after infecting cancer cell in addition to dying naturally. Note that we assume that each cancer cell needs one virus to infect it, and thus, *κ* in the third equation in (1) is equal to 1.

Based on the aforementioned assumptions, we obtain the following system of PDEs:

(1) $$\begin{array}{c} \frac{\partial x}{\partial t}=D_{1}\nabla^{2}x+r_{1}x-\beta xv,\\ \frac{\partial y}{\partial t}=D_{1}\nabla^{2}y+\beta xv-\delta y,\\ \frac{\partial v}{\partial t}=D_{2}\nabla^{2}v+b\delta y-\kappa \beta xv-\alpha v, \end{array}$$

with the following homogenous initial conditions and boundary conditions:

(2) $$\begin{array}{c} x\left(r,0\right)=x_{0}, \end{array}$$

(3) $$\begin{array}{c} \frac{\partial x}{\partial r}\left(r_{b},t\right)=\frac{\partial x}{\partial r}\left(\frac{r_{b}}{\sqrt{\text{BVF}}},t\right)=0, \end{array}$$

The physical variables in our model consist of the following: *x* = *x*(*r*, *t*) is the density of uninfected tumor cells,  *y* = *y*(*r*, *t*) is the density of infected tumor cells, and *v* = *v*(*r*, *t*) is the density of free viruses.

Table 1 gives a summary of all the model parameters and their description, values, and units.

It should be noted that our model is similar to the one in [12], where the new addition of terms involves the reduction of free virus density due to the infection of tumor cells. We have also incorporated the spatial variation of the variables due to the diffusion of the drug and cancer cells.

### 2.2. Numerical Solution

In this section, we solve the model (1) with initial conditions (2) and boundary conditions (3) numerically. Additionally, we vary the parameters to determine the key parameters that can improve the outcomes of virotherapy treatment. We discretize in space and time and use the fourth-order Runge-Kutta Method [19] for the time discretization and the finite difference method [20] for the space discretization. The numerical simulations are carried out by using the values of the parameters as given in Table 1. The initial conditions and boundary conditions are chosen as follows:

(4) $$\begin{array}{c} x\left(r,0\right)=0.5\times 10^{6}, \end{array}$$

(5) $$\begin{array}{c} \frac{\partial y}{\partial r}\left(r_{b},t\right)=\frac{\partial y}{\partial r}\left(\frac{r_{b}}{\sqrt{\text{BVF}}},t\right)=0, \end{array}$$

where *r*_*b*_ = 0.01 and BVF = 0.05. Moreover, the initial conditions have the unit cell/mm^3^ (for *x* and *y*) or virus/mm^3^ (for *v*). The no flux boundary has the unit (cell/mm^3^)/mm.

In the numerical simulations, we calculate the ratio of the viable (uninfected) tumor mass to its initial mass *M*_0_ (normalized cancer mass). We integrate the density *x* at each time step over the cylindrically symmetric domain surrounding the blood vessel as follows:

(6) $$\begin{array}{c} f_{x}\left(t\right)=\frac{1}{M_{0}}\int_{0}^{2\pi }\int_{r_{b}}^{r_{b}/\sqrt{\text{BVF}}}x\;r\;dr\;d\theta =\frac{2\pi }{M_{0}}\int_{r_{b}}^{r_{b}/\sqrt{\text{BVF}}}x\;r\;dr, \end{array}$$

where $V=\pi \left[{\left(r_{b}/\sqrt{\text{BVF}}\right)}^{2}-{\left(r_{b}\right)}^{2}\right]$ and *M*_0_ = *x*_0_*V*.

**Figure 1:**
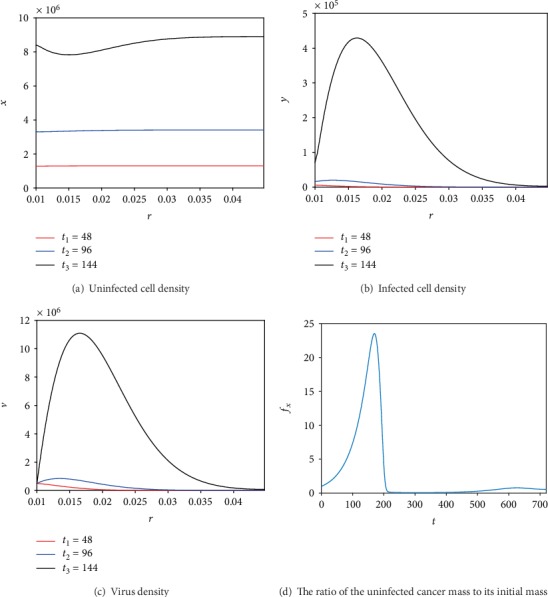
Numerical simulations of (1) with the conditions (4) and (5) and parameter values from Table 1 (*t* is given in hours). For (a)–(c), the variables are plotted after the second, fourth, and sixth day of the treatment as indicated in the legend. In (d), the temporal evolution curve of the ratio of the uninfected cancer mass to its initial mass is plotted for a treatment that lasted 720 hours (30 days).

First, we simulate the model and display the changes in the density of the variables for different times as shown in Figures 1(a)–1(c). The plots illustrate the densities *x*, *y*, and *v*, respectively, after the second, fourth, and sixth day of the treatment, where the treatment is administered for 30 days. The success of the treatment is judged based on its capability to eliminate tumor cells. Our focus then is on the examination of the viable tumor cells and what will happen to them as other cells deteriorate and eventually die. The temporal variation curve of the ratio of the viable tumor mass to its initial mass for the whole treatment is shown in Figure 1(d). The simulation shows that for 30 days of treatment, the tumor grows in the beginning of the treatment. Then, after approximately seven days, the viruses overcome this growth and limit the tumor mass. Afterwards, the tumor mass decreases to reach small values (approximately 7% of its original mass). This reduction will result in a decrease in the viruses, and thus, we observe a recurrence of the tumor, which will in turn cause an increase for the viruses. The latter will again reduce the tumor. This oscillatory behavior where cancer decreases, regrows, and decreases was also observed in the mathematical models studied in [21, 22]. Finally, after approximately 30 days, the tumor mass is reduced to about 55% of its original mass.

#### 2.2.1. Parameter Analysis

In this section, we discuss the changes in the viable cancer mass by varying the parameters in the model. The focus here is on the key parameters that are relevant to the oncolytic viruses. Specifically, we vary the viral infection rate (*β*), the virus burst size (*b*), and the clearance rate of viruses (*α*). All the other parameters remain the same as in Table 1.

**Figure 2:**
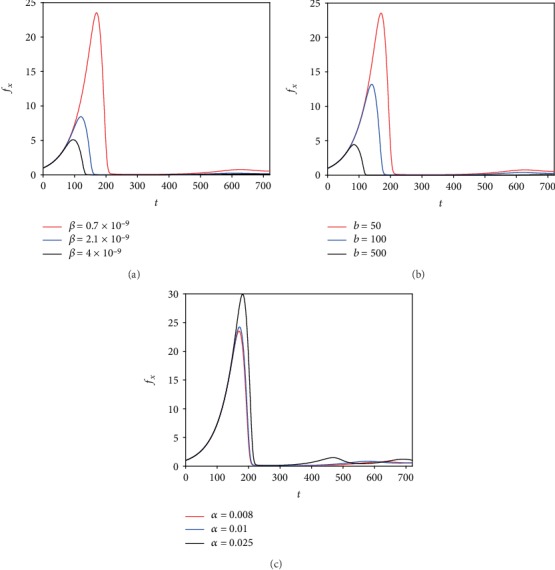
The ratio of the uninfected cancer mass to its initial mass of (1), (4), and (5) is plotted for different values of the parameters as shown in the legend (the other parameters are taken from Table 1).

**Figure 3:**
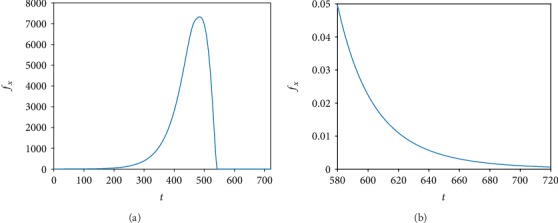
Numerical simulations of (1) with the conditions (4) and (5) and parameter values from Table 1 with *b* = 2. (b) is the same as (a) for *t* = 580 − 720 hours.

To begin with, we vary *β*, which is the viral infection rate.  Figure 2(a) illustrates the time variation curves of the ratio of the viable tumor mass to its initial mass by varying the values of *β* as indicated in the legend. We found that the increase in the viral infection rate reduces *f*_*x*_. In addition, the treatment takes a shorter time to control the tumor mass and this is the desired result of virotherapy. Next, we vary the virus burst size (*b*).  Figure 2(b) shows the temporal evolution curves of the ratio of the viable tumor mass to its initial mass with varying values of *b*. It should be pointed out that the increase in the virus burst size means that there are more new viruses resulting from the lysis of infected tumor cells. Thus, we found that the largest value of *b* leads to a better outcome of virotherapy. Also, as *b* increases, new viruses are produced at a large number. This may take a long time and thus affects the death rate of infected cells by making it slower. This case is not considered in the simulations as *δ* is assumed to be constant for all values of *b*. On the other hand, if we decrease the value of *b* to a very small number, for example, *b* = 2, then virotherapy becomes similar to chemotherapy where it is consumed by cancer cells. Thus, the oscillation disappears as shown in Figure 3 unlike Figure 2(b). This shows that virotherapy alone (even with large values of *b*) is insufficient as there is always the risk of growing back of small residuals as shown in Figure 2(b). As for the viral clearance rate, we note in Figure 2(c) that the ratio of the viable cancer mass to its initial mass increases for large values of *α*. This is as a result of insufficient viruses to inhibit tumor growth.

From the simulations above, we conclude that virotherapy alone is not sufficient to eradicate all tumor cells. This is because at the end of the simulation in Figures 1(d) and 2, the normalized cancer mass becomes drastically small but then grows back. This means that it is not eradicated. We consider cancer to be eradicated if the normalized mass becomes very small and does not grow back. Since this did not happen, it is necessary to incorporate another treatment with virotherapy. The next section will investigate radiovirotherapy as a second phase of the treatment, and thus, the spatiotemporal dynamics of tumor cells under combination treatment between virotherapy and radiotherapy will be considered.

## 3. Phase II: Radiovirotherapy Treatment

After Phase I of the treatment has been implemented, Phase II which consists of radiovirotherapy commences. In this way, the initial treatment starts with just virotherapy, which continues until time *t*_*r*_. Thereafter, radiotherapy is introduced to supplement the virotherapy treatment. This is done because the numerical results in the previous section show that virotherapy alone is not enough to eliminate cancer.

Here, we provide a model that includes a combination of treatments against tumor, and this approach incorporates the use of virotherapy combined with radiotherapy. In particular, we extend the virotherapy model (1) by including the effects of radiotherapy on both types of tumor cells. Consequently, we insert a new physical variable that represents the density of all tumor cells that are irreparably damaged by radiation and these cells are removed from the body (*u* = *u*(*r*, *t*)). Thus, the mathematical model consists of the following system of four PDEs with parameters as described in Table 1:

(7) $$\begin{array}{c} \frac{\partial x}{\partial t}=D_{1}\nabla^{2}x+r_{1}x-\beta xv-a_{1}R\left(t\right)x,\\ \frac{\partial y}{\partial t}=D_{1}\nabla^{2}y+\beta xv-\delta y-a_{2}R\left(t\right)y,\\ \frac{\partial u}{\partial t}=a_{1}R\left(t\right)x+a_{2}R\left(t\right)y-\gamma u,\\ \frac{\partial v}{\partial t}=D_{2}\nabla^{2}v+b\delta y-\beta xv-\alpha v. \end{array}$$

After solving the first model (1) numerically at each time step until time *t* = *t*_*r*_ (Phase I ends), and these solutions become initial values for the second model (Phase II starts), thus, we have the following initial conditions for (7):

(8) $$\begin{array}{c} x\left(r,t_{r}\right)=x_{r},\\ y\left(r,t_{r}\right)=y_{r},\\ u\left(r,t_{r}\right)=u_{r},\\ v\left(r,t_{r}\right)=v_{r}, \end{array}$$

where *x*_*r*_, *y*_*r*_, and *v*_*r*_ are solutions of model (1) at time *t* = *t*_*r*_.

The protocol for administering radiation must be chosen before solving (7) numerically. There are several kinds, which include constant, linear control (proportional to the size of tumor), feedback control (proportional to the ratio of cancer to healthy tissue), and periodic deliveries [23]. In this case, we examine three types of deliveries: constant, decaying, and periodic radiation (similar to the model in [14]). In practice, constant radiation can be carried out by using a temporary brachytherapy, in which radioactive material is placed inside a catheter for a specific period of time then it is withdrawn from the body. Thus, the radioactive material stays in the body and omits a constant dose of radiation without decaying, and then, it is withdrawn. Regarding the decaying radiation, it can be employed by a permanent brachytherapy, where radioactive material is implanted in the tumor site; then after several months, the radiation dose emitted from the source decreases and vanishes. Thus, in this case, radiation decays over time. Regarding the periodic radiation, it can be executed by an external beam radiation, which uses a machine to direct high-energy rays towards the tumor site [24]. In this way, three modes of *R* (*t*) are utilized as follows:

- *R* (*t*) = *R*, constant
- *R* (*t*) = *β*_1_*e*^−*α*_1_*t*^, decay
- *R* (*t*) = *β*_2_ + *α*_2_ sin*ωt*, periodic

### 3.1. Numerical Solution

In this section, we first solve the model (1) with conditions ((4) and (5)) for Phase I. The solution will be calculated for 5 days (*t*_*r*_ = 120 hours), unless otherwise stated. After that, we solve model (7) numerically (using the same numerical method as (1)) with the boundary conditions given in (5) and initial conditions (8) for Phase II. The initial condition for *u* is chosen to be *u*(*r*, *t*_*r*_) = 0. The values of the parameters are as given in Table 1. In all simulations, we calculate *f*_*x*_ (*t*) from (6), where the initial mass is *M*_0_ = *x*_*r*_*V* (the mass of the uninfected cancer cells at the beginning of Phase II). We also discuss three protocols for the administration of radiation, which are constant, decaying, and periodic radiation as follows:

(9) $$\begin{array}{c} R\left(t\right)=\left\{\begin{array}{cc} 2, & \text{constant},\\ 2\;e^{-0.01\;t}, & \text{decay},\\ 1+\sin2t, & \text{periodic}. \end{array}\right. \end{array}$$

The goal of comparing the result of these three modes of radiation is that oncologists might prefer a certain kind of radiation delivery for a specific reason, perhaps for being practical. Thus, in the following simulations, we give an insight about the outcome of each kind of protocol by using mathematical modeling. Of course, all of these results need validation by experiments and clinical trials.

**Figure 4:**
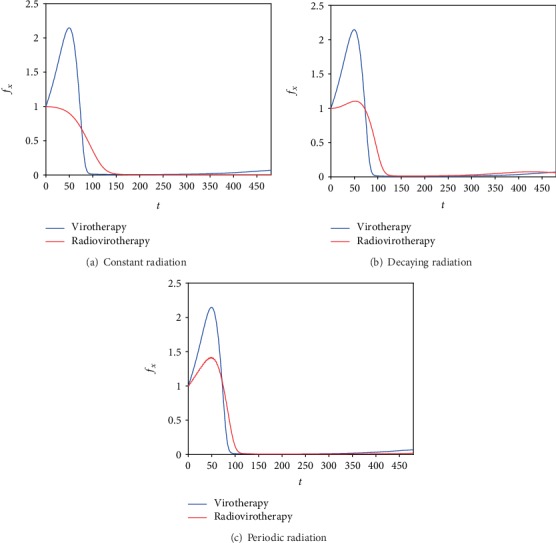
The ratio of the uninfected cancer mass to its initial mass is plotted after day 5 from the beginning of virotherapy alone. The red curve represents radiovirotherapy with three types of radiation delivery: constant, decaying, and periodic delivery, whereas the blue curve means that virotherapy continues without radiotherapy.

Figure 4 represents time evolution of the viable cancer mass to its initial mass for constant, decaying, and periodic radiation doses, respectively (red curves). The radiovirotherapy began after 5 days of virotherapy. For comparison, the blue curves represent the result for virotherapy alone (after giving virotherapy for 5 days, radiation is not introduced and instead virotherapy continues until the end of the simulation). The numerical results show that the combination of radiotherapy with virotherapy is more effective in reducing the mass of the cancer than virotherapy alone. Specifically, constant radiation combined with virotherapy eliminates cancer unlike virotherapy. This shows that the effects of radiotherapy on the tumor depend on the type of radiation delivery.

**Figure 5:**
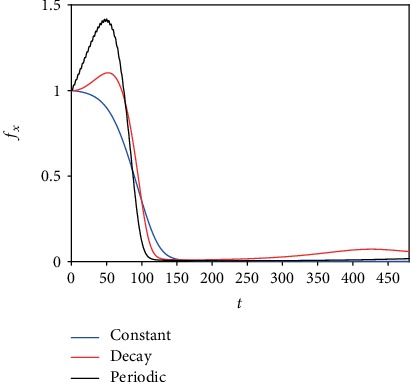
The effects of three different modes of radiation on the ratio of the uninfected cancer mass to its initial mass.

Figure 5 shows the numerically calculated value of the ratio of the viable cancer mass to its initial mass with virotherapy in combination with three types of radiation delivery as indicated in the legend. In the beginning of the simulation, the constant and decaying cases both have the same result (since *R*(*t*) at the beginning is equal to 2); then, the constant case has a better result (since for the decaying radiation *R* (*t*) becomes less than 2). For the periodic case, it eventually catches up with the constant case after *R* (*t*) reaches the value of 2. The tumor decreases to approximately 1% of its original mass in about 6 days and continues decaying with constant radiation. For the periodic and decaying radiation, the tumor decreases to small values after approximately 6 days but then regrows to larger values for the decaying radiation compared to the periodic radiation.

**Figure 6:**
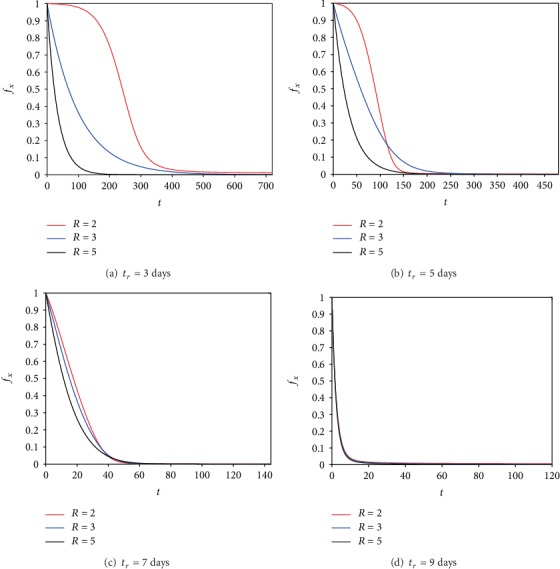
Plots of the ratio of the uninfected cancer mass to its initial mass with different doses of constant radiation as indicated in the legend and four different timings *t*_*r*_ for administering the radiation.

These results demonstrated that the dose of radiation is a critical factor that affects the outcomes of radiotherapy and virotherapy [9]. Therefore, we performed numerical simulations to show the effects of the different doses of constant radiation at four different timings (*t*_*r*_) when radiation is administered.  Figure 6 illustrates the start of radiotherapy after the third, fifth, seventh, and ninth days (respectively) from the beginning of virotherapy.  Figure 6(a) represents Phase II after 3 days of virotherapy (that is when cancer was growing as shown in Figure 1(d)). The simulation in Figure 6(a) shows that cancer is eradicated in a short time for a high dose of radiation. If we increase *t*_*r*_ to reach 5 days, then also high radiation has the best result but the different doses of radiation begin to have a similar effect on the normalized cancer mass. Now when *t*_*r*_ = 7 (that is when cancer reaches its maximum value in Figure 1(d)), the three doses of radiation have similar results and eradicate cancer in a shorter time than the previous case. These three doses have an almost similar result in eradicating cancer in a shorter time than the previous case if *t*_*r*_ = 9. These simulations show that, if Phase II starts early (*t*_*r*_ < 7), a high dose of radiation is needed to overcome the growth of cancer. On the other hand, any dose will be sufficient if Phase II starts after the seventh day of virotherapy as cancer starts to decrease from the previous treatment.

## 4. Discussion and Future Research

Oncolytic viruses are a novel type for cancer treatment. These viruses are currently being delivered alone or as a part of a combination treatment regime with conventional therapies such as radiotherapy. Research studies indicate that using therapeutic viruses in combination with radiation therapy is more effective than making use of virotherapy alone. As this field of research is promising and needs more developing, in this paper, we introduced systems of PDEs to simulate two phases of treatments, which are virotherapy and radiovirotherapy. In phase I, virotherapy is given continuously for a certain period of time, which may be clinically achieved through nanovectored delivery. This kind of delivery is chosen since in vivo experiments of a breast cancer mouse model with different kinds of drug delivery show that there is a threefold increase in response by using nanovectored drug compared to a free drug delivery [16]. To model this phase, we introduced a system of PDEs illustrating the spatiotemporal dynamics between virotherapy and infected and uninfected cancer cells. The numerical simulations were carried out for different values of the parameters related to virotherapy, namely, burst size and infection and clearance rates of the virus. These solutions showed that virotherapy alone is not enough to eliminate cancer. Thus, we introduced phase II of the therapy, which is radiovirotherapy. To model this phase, we extended the previous system by incorporating radiotherapy alongside virotherapy. Specifically, we added a fourth equation representing damaged cancer cells due to radiation. Phase II was studied numerically, which showed that radiovirotherapy leads to a complete eradication of a tumor provided that the radiation delivery is constant. It was also concluded that a high dose of continuous radiation with an early start of Phase II leads to killing cancer cells in a short time. Although this is a desired result, dose escalation is limited because radiation can cause severe damage to healthy tissues [25].

Future research could include an investigation into the damage caused from radiation on normal body tissue. Another research point could be considering different delivery method for viruses, for example, giving the virus in cycles [26]. This can be analyzed and compared to our results where viruses are delivered continuously for a certain period of time. Finally, validating these models by experiments and patients' data could be beneficial for oncologists to predict the outcomes of treatments without patients' suffering.

## Figures and Tables

**Table 1:** The parameters and their description, values, and units for models (1) and (7).

Parameter	Description	Value	Units	Reference
*D*_1_	Diffusion coefficient of tumor cells	10^−8^	mm^2^*/*h	Estimated
*r*_1_	Tumor growth rate	0.02	1/h	[18]
*β*	Infection rate of the virus	7/10 × 10^−9^	mm^3^*/*h virus	[18]
*δ*	Death rate of infected tumor cells	1/18	1/h	[18]
*D*_2_	Diffusion coefficient of viruses	5 × 10^−7^	mm^2^*/*h	Estimated
*b*	Burst size of free virus	50	Viruses/cell	[18]
*κ*	Consumption rate of the virus	1	Virus/cell	Estimated
*α*	Clearance rate of the virus	0.008	1/h	Estimated
*a*_1_	Rate of which the uninfected tumor cells become irreparably damaged	0.01	1/h	Estimated
*a*_2_	Rate of which the infected tumor cells become irreparably damaged	0.01	1/h	Estimated
*γ*	Death rate of damaged cells	0.01	1/h	Estimated
*r*_*b*_	Radius of the blood vessel	0.01	mm	Estimated
BVF	Blood volume fraction	0.05	—	[16]
*v*_0_	Value of *v* at the blood vessel wall	0.5 × 10^6^	Viruses/mm^3^	Estimated
*t*_*r*_	The time where Phase II begins	120	h	Estimated

## Data Availability

The data used to support the findings of this study are included within the article.
